# Signaling Circuits and Regulation of Immune Suppression by Ovarian Tumor-Associated Macrophages

**DOI:** 10.3390/vaccines3020448

**Published:** 2015-05-29

**Authors:** Martin J. Cannon, Debopam Ghosh, Swetha Gujja

**Affiliations:** 1 Department of Microbiology and Immunology, University of Arkansas for Medical Sciences, 4301 West Markham, Little Rock, AR 72205, USA; E-Mail: DGHOSH@uams.edu; 2 Department of Internal Medicine, University of Arkansas for Medical Sciences, 4301 West Markham, Little Rock, AR 72205, USA; E-Mail: SGujja@uams.edu

**Keywords:** ovarian cancer, macrophages, dendritic cells, regulatory T cells, indoleamine 2,3-dioxygenase, aryl hydrocarbon receptor, c-KIT, STAT3

## Abstract

The barriers presented by immune suppression in the ovarian tumor microenvironment present one of the biggest challenges to development of successful tumor vaccine strategies for prevention of disease recurrence and progression following primary surgery and chemotherapy. New insights gained over the last decade have revealed multiple mechanisms of immune regulation, with ovarian tumor-associated macrophages/DC likely to fulfill a central role in creating a highly immunosuppressive milieu that supports disease progression and blocks anti-tumor immunity. This review provides an appraisal of some of the key signaling pathways that may contribute to immune suppression in ovarian cancer, with a particular focus on the potential involvement of the c-KIT/PI3K/AKT, wnt/β-catenin, IL-6/STAT3 and AhR signaling pathways in regulation of indoleamine 2,3-dioxygenase expression in tumor-associated macrophages. Knowledge of intercellular and intracellular circuits that shape immune suppression may afford insights for development of adjuvant treatments that alleviate immunosuppression in the tumor microenvironment and enhance the clinical efficacy of ovarian tumor vaccines.

## 1. Introduction

The history of tumor vaccination has reported a series of impressive successes in treating cancer in animal models, but these successes have not translated to clinical efficacy for human patients. There are a number of fairly obvious reasons for this state of affairs. First, human patients are far more diverse and far less predictable than highly optimized experimental systems with inbred mice. Second, the primary endpoints of phase I clinical trials are determination of safety and maximum tolerated dose, and thus phase I trials traditionally enroll subjects with advanced disease and significant tumor burden. The extensive comorbidities prevalent in late stage disease make protocol adherence and completion a challenge, render immune responses inconsistent, and clinical responses elusive. As tumor vaccines take time to stimulate effective anti-tumor immunity through repeated dose regimens, there has been increasing recognition that phase I clinical trials should be conducted whenever possible in patients with early stage disease (but significant risk of progression), minimal tumor burden, limited comorbidities, and a high level of immune competence. The primary endpoint is still establishment of safety, but there is an improved opportunity that secondary endpoints of immune response and clinical response (delayed time to recurrence/progression) may be met. The third barrier to the clinical success of tumor vaccination is the burgeoning recognition that immune suppression in the tumor microenvironment presents a formidable barrier to the achievement of clinical responses, no matter how immunogenic the vaccine, or how well chosen the subject population.

Ovarian tumors avail themselves of multiple mechanisms of immune evasion, thus, blunting the efficacy of therapeutic vaccination or immunotherapy. Regulatory T cells (Treg) are recruited to ovarian tumors by the chemokine CCL22 (which is highly expressed by ovarian tumors), and the presence of Treg confers immune privilege and is associated with a poor prognosis and increased mortality [1,2]. Further mechanisms include expression of PD-L1, which can promote T cell anergy and apoptosis through engagement of PD-1 expressed by effector T cells, and has been associated with increased morbidity and mortality in patients with ovarian cancer [3]. Of particular interest, indoleamine 2,3-dioxygenase (IDO) expression also correlates with poor outcomes in ovarian cancer [4,5]. This is notable because IDO may play a pivotal role in the balance between Treg and Th17 differentiation, and IDO activity is a known mechanism of immune suppression by ovarian tumor-associated macrophages [6]. In this brief review, we explore some of the key pathways that may be operative in the ovarian tumor microenvironment, with a particular focus on the central role that may be played by tumor-infiltrating macrophages/dendritic cells (DC). An understanding of the regulatory circuitry and cellular interactions that control immune suppression in the tumor microenvironment may yield opportunities for adjuvant treatments that counterbalance immune suppression and promote vaccine-induced anti-tumor immunity. All the mechanisms under discussion are well known in various contexts, but an integrated picture of the immunoregulatory circuitry has yet to be established for ovarian cancer.

## 2. Immune Suppression by Ovarian Tumor-Associated Macrophages

CD14+ cells are abundant in human ovarian tumor ascites, and represent the major population of myeloid suppressor cells in ovarian cancer [6,7]. These cells display overlapping characteristics of macrophages, dendritic cells and myeloid-derived suppressor cells (MDSC), with a CD14+CD11b+CD11c+ phenotype, and consistent expression of both PD-1 and PD-L1 [6,8]. Ovarian tumor-associated CD14+ myeloid cells are chemo-attractive for CD4+ T cells, and are strongly immunosuppressive for dendritic cell-stimulated tumor antigen-specific T cell responses [6]. The major mechanisms of immune suppression include indoleamine 2,3-dioxygenase (IDO) activity and secretion of IL-10 [6]. Related studies have described similarly immunosuppressive macrophage/DC/MDSC phenotypes in mouse models of ovarian cancer, and have shown that myeloid cells are the dominant infiltrating component in late stage ovarian tumors [8,9]. Clinical analysis has indicated that evidence of M2 polarization of ovarian tumor-associated macrophages, including high CD163 expression and high ascites IL-6 and IL-10 levels, correlates with poor clinical outcomes [10], although in many patients gene expression profiles revealed a mixed phenotype that was not amenable to M1/M2 assignment.

### 2.1. A Central Role for STAT3

**Figure 1:**
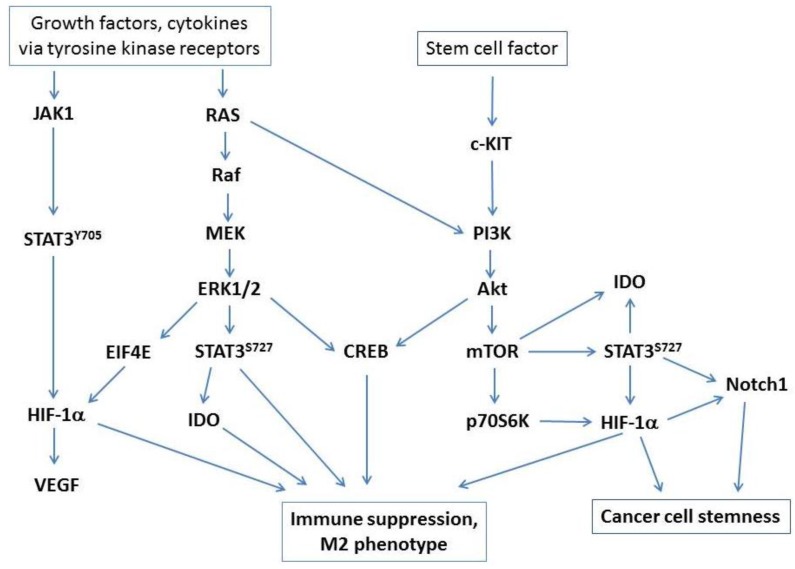
Key signaling pathways that drive immune suppression by tumor-associated macrophages. Major pathways activated via tyrosine kinase receptors or c-KIT/PI3K/Akt signaling (activated by stem cell factor, which is abundantly expressed in ovarian cancer) converge on STAT3 and CREB activation and induction of HIF-1α under hypoxic conditions.

STAT3 signaling has long been known to play a critical role in immune tolerance and inhibition of tumor immune surveillance [11,12], and multiple studies point to a central role for STAT3 signaling as a key driver of immune suppression by tumor-associated macrophages [13] and M2 polarization in ovarian cancer [14]. STAT3 can be activated via the JAK/STAT3(pY705) pathway, which induces HIF-1α and VEGF expression. The MEK/ERK MAPK pathway drives STAT3(pS727) activation, and the c-KIT/PI3K/AKT/mTOR pathway induces STAT3(pS727) phosphorylation, leading to expression of IDO and HIF-1α. The PI3K/AKT/CREB axis may also be a predominant signaling pathway driving the M2 phenotype of ovarian tumor-associated CD14+ cells. AKT is known to be a potent activator of CREB through a PI3K-dependent mechanism [15], and various studies have pointed to a central role for CREB in driving macrophage polarization through upregulation of M2-specific genes [16,17]. CREB is widely recognized to induce IL-10 expression, which is elevated in ovarian tumor ascites and is a mechanism of suppression by ovarian tumor-associated CD14+ cells [6]. Key signaling pathways in macrophage regulation of tumor-associated immune suppression are presented in Figure 1.

### 2.2. Hypoxia, Immune Suppression and Tumor Progression

Hypoxia is intimately involved in regulation of tumor-associated CD14+ cell function, immune suppression and tumor progression. HIF-1α regulates myeloid-derived suppressor cell function [18] and macrophage expression of HIF-1α contributes to suppression of T cell responses [19]. Of particular significance, (i) macrophages inhibit T cell proliferation under hypoxia; (ii) T cell suppression in hypoxia is HIF-1α dependent; and (iii) hypoxia induces IDO expression, which can be reproduced by treatment with the hypoxia mimetic 2,2-dipyridyl under normoxic conditions [19]. These studies provide a direct link between hypoxia and IDO activity, which is a known mechanism of immune suppression of DC-stimulated CD4+ T cell responses by ovarian tumor-associated CD14+ macrophages [6]. An important recent study found that hypoxia-induced Semaphorin 3A acts as an attractant for tumor-associated macrophages and further showed that prevention of hypoxic migration abated macrophage immune suppression and promoted anti-tumor immunity [20]. From a clinical perspective, it is perhaps unsurprising that hypoxia and tumor-associated macrophage infiltration correlate with reduced overall survival rates [21].

HIF-1α expression is also central to tumor progression and neo-angiogenesis. Hypoxia induces expression and activation of STAT3, whereas oxygenation inhibits STAT3 activation and limits growth of human ovarian tumor xenografts in mice [22]. Furthermore, inhibition of HIF-1α expression in ovarian tumor cells slowed formation of tubular networks by human umbilical vein endothelial cells, suggesting inhibition of tumor-associated neo-angiogenesis [23]. Hypoxia and HIF-1α are also key to maintenance of cancer cell stemness via Notch1 signaling [24,25], and hypoxia induces expression of genes associated with stemness in ovarian cancer [26]. In a mouse model of pancreatic cancer, STAT3 activation in monocytic suppressor cells increases the frequency of aldehyde dehydrogenase-positive cancer stem cells [27], indicating that STAT3 activation in myeloid cells is not only central to immune suppression, but also plays a role in tumor cell stemness, survival and progression. A recent study showed that STAT3 and HIF-1α form transcription factor complexes that drive target gene expression, providing a direct cooperative link between STAT3 and HIF-1α in tumor progression [28]. It is not known whether similar mechanisms of transcriptional cooperation apply in ovarian tumor-associated macrophages/DC, but it will be apparent that targeting of STAT3 and/or HIF-1α could be of manifold benefit in alleviating tumor-associated immune suppression and limiting tumor cell survival and disease progression.

## 3. Regulation of IDO Expression and Treg Differentiation

IDO is a key regulator of Treg/Th17 immunity [29,30], and is a known mechanism of immune suppression by tumor-associated ascites CD14+ myeloid cells from ovarian cancer patients [6]. The clinical relevance of these observations is clearly shown by the association of IDO expression with increased morbidity and mortality in patients with ovarian cancer [4,5]. Although the mechanisms by which IDO expression and function are regulated in ovarian tumor-associated CD14+ myeloid cells have not been elucidated, various regulatory pathways have been described in other settings, any or all of which could be important for immune regulation in ovarian cancer.

### 3.1. Stem Cell Factor and c-KIT Signaling

IDO expression can be blocked by inhibitors of c-KIT or mTOR (downstream of the c-KIT/PI3K/AKT pathway), with resultant enhancement of anti-tumor T cell responses [31], but the potential role of PI3K/AKT/CREB signaling or STAT3 activation has not been addressed. The ligand for c-KIT is stem cell factor (SCF), which is secreted by ovarian tumors [32,33,34] and is present at high levels in tumor ascites [35], thus the question of whether IDO expression can be induced by c-KIT signaling in tumor-associated macrophages has immediate relevance for immune regulation in the ovarian tumor microenvironment and for the pathogenesis of disease.

### 3.2. The IDO-AhR Relationship

Recent studies have revealed a complex relationship between IDO function and AhR on myeloid cells and T cells. Binding of the AhR promotes generation of regulatory T cells [36,37,38] and AhR ligand-specific interactions may control the balance between Treg and Th17 differentiation [39,40]. The tryptophan catabolite kynurenine produced by IDO is a natural ligand for AhR [41,42], thus creating a mechanism by which IDO induces Treg differentiation. These observations support an innovative model in which SCF binds c-KIT expressed by ovarian tumor ascites CD14+ cells, resulting in IDO expression and Treg differentiation through kynurenine production and binding to AhR. Two complementary pathways downstream of AhR may be operative in driving Treg differentiation. First, activation of AhR expressed by T cells can induce Foxp3 expression, in part through demethylation of the Foxp3 promoter, which is accompanied by increased methylation of the IL-17 promoter, thus limiting IL-17 expression and inhibiting development of Th17 cells [43]. Second, activation of AhR in myeloid cells can induce aldehyde dehydrogenase (ALDH) [44], resulting in production of retinoic acid, which subsequently binds RA receptors in T cells. The RA/RA receptor complex drives Foxp3 expression in combination with other transcription factors, including Smad3 [45].

**Figure 2:**
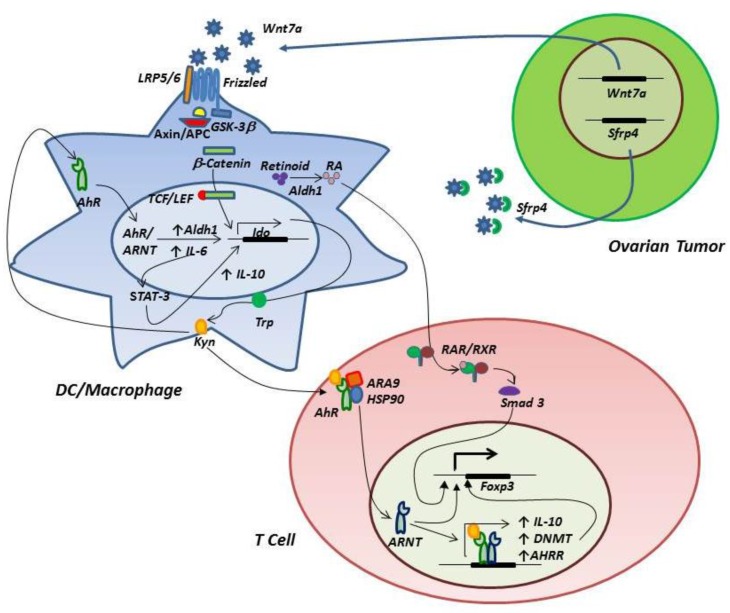
A model of IDO/AhR/wnt/β-catenin circuitry in the ovarian tumor-associated microenvironment. Ovarian tumor expression of wnt7a leads to engagement of LRP5/6 and frizzled receptors on tumor-associated myeloid cells, resulting in release of β-catenin from the axin/APC/GSK3β complex, followed by translocation to the nucleus, association with TCL/LEF and expression of IDO. Expression of wnt7a is negatively regulated by SFRP4. Kynurenine produced by IDO binds myeloid cell AhR, resulting in association of AhR with its nuclear translocator (ARNT) and subsequent transcriptional activation of IDO, ALDH1 and IL-6 expression. ALDH1 contributes to catabolism of retinoid to retinoic acid (RA), and IL-6 drives to a positive feedback loop involving STAT3 activation, IDO expression, kynurenine production and AhR signaling. STAT3 activation also shapes an M2 macrophage phenotype characterized by high IL-10 production. RA binds T cell retinoic acid receptors (RAR/RXR) and induces Foxp3 expression and Treg differentiation. Kynurenine binds T cell AhR, which dissociates from ARA9/HSP90, binds ARNT and induces expression of Foxp3, DNA methyl transferase (DNMT, which demethylates the Foxp3 promoter), IL-10 and the AhR repressor (AHRR), which provides negative feedback for inhibition of AhR signaling.

In a variation on this theme, but also involving AhR, a positive feedback loop encompassing IL-6 expression and STAT3 has recently been described in various tumor cells, including ovarian cancer [46]. IDO production of kynurenine activates AhR, resulting in IL-6 expression, which in turn drives IDO expression via STAT3 activation, thus completing an autocrine loop. Analysis of the IDO1 promoter revealed STAT3 binding sites, and inhibition of STAT3 phosphorylation could reduce IDO mRNA and protein expression and diminish IDO enzyme activity. Similarly, siRNA knockdown of IL-6 inhibited IDO expression in SKOV3 ovarian cancer cells and reduced the ability of tumor cells to suppress T cell responses in mixed lymphocyte reactions. Figure 2 models key signaling circuits involving IDO, AhR and wnt/β-catenin (considered in the following section).

### 3.3. Signaling through the Wnt/β-Catenin Pathway

Other pathways that may regulate IDO expression include wnt/β-catenin signaling, which is active in ovarian cancer and has been implicated in carcinogenesis and tumor progression [47,48]. Wnt7a expression in ovarian cancer has been associated with advanced stage and high grade, and may be a significant prognostic factor in ovarian cancer [49]. Although ovarian tumor cells are known to produce Wnt7a, it should be noted that tumor-associated macrophages may also be an important source of Wnt ligands that contribute to tumor progression [50]. In the absence of wnt binding to receptors, the default setting of the canonical wnt/β-catenin pathway is off, and cytoplasmic β-catenin is targeted for proteasomal degradation by a complex of axin, adenomatous polyposis coli (APC) and glycogen synthase kinase 3β (GSK3β). Depressed levels of β-catenin allow binding of the repressor molecule Groucho to TCF/LEF transcription factors, thus inhibiting expression of target genes associated with an anti-inflammatory phenotype in macrophages [51] and dendritic cells [52]. The pathway is activated when wnt binds one of several cell surface receptors (including frizzled and low density lipoprotein receptors LRP5 and LRP6), leading to inactivation of the β-catenin degradation complex and release of β-catenin to the nucleus, where it displaces Groucho from the TCF/LEF transcription complex. Wnt signaling can be competitively inhibited by secreted frizzled-related protein 4 (SFRP4), the loss of which has been associated with poor outcomes in ovarian cancer patients [53] and decreased responsiveness of ovarian cancer cells to cisplatin *in vitro* [54].

From an immunological perspective, activation of β-catenin can regulate the balance between Treg and Th17 T cell responses, with β-catenin signaling favoring expression of IL-10, TGFβ and aldehyde dehydrogenase enzymes (involved in vitamin A metabolism and retinoic acid production), thus promoting Treg induction [52]. Since p38 MAPK signaling can enhance β-catenin activity by inactivation of GSK3β [55,56], inhibition of p38 may result in diminished levels of β-catenin, favoring a pro-inflammatory Th17-biased T cell response. Of particular interest, the IDO1 promoter contains TCF/LEF-binding domains and wnt-activated LEF-1 increases IDO1 promoter activity [57], thus providing a potential mechanism by which p38 and wnt can regulate IDO activity, which likely sits at the fulcrum of Treg/Th17 regulation. In this context, it is notable that p38 blockade almost totally inhibits IDO activity in human dendritic cells [58].

## 4. Regulation of Co-Inhibitory Molecule Expression

Clinical trials have shown that antibody blockade of PD-1/PD-L1 can lead to remarkable clinical responses in cancer patients [59,60,61], resulting in FDA approval of lambrolizumab (anti-PD-1) in 2014, with several other promising candidates likely to gain approval in the near future. Ovarian tumor-associated macrophages invariably express PD-L1 (B7-H1) and PD-1 [6,8], and suppress T cell responses. PD-L1 expression is known to correlate with poorer clinical outcomes in ovarian cancer [3], and thus elucidation of the mechanisms by which PD-L1 expression is regulated may have profound translational and clinical impact. Recent studies have shown that PD-L1 expression is regulated in a STAT3-dependent manner, with chromatin immunoprecipitation assays revealing that STAT3 directly binds the PD-L1 promoter [62]. It will be important to determine whether STAT3 phosphorylation correlates with PD-L1 expression, and further determine whether treatment of ovarian tumor ascites CD14+ cells with small molecule inhibitors of STAT3 leads to reduced PD-L1 expression.

A related co-inhibitory molecule, B7-H4, is also expressed by ovarian tumor-associated macrophages, and B7-H4^+^ macrophages (but not primary ovarian tumor cells) suppress tumor antigen-specific T cell responses [63]. B7-H4 expression by ovarian tumor-associated macrophages (but not tumor cell B7-H4) correlates with infiltrating Treg numbers, and macrophage B7-H4 expression is associated with poor clinical outcomes [64]. Treg can induce B7-H4 expression via an autocrine loop involving macrophage expression of IL-10 [65], but there is no information on the signaling pathways by which B7-H4 expression is controlled. It is probable that B7-H4 is at least in part regulated by JAK/STAT3 signaling downstream of IL-6 and IL-10 receptors in ovarian tumor-associated macrophages, but this has yet to be formally tested.

## 5. Potential Therapeutic Interventions that Target Ovarian Tumor-Associated Macrophage Signaling and IDO Expression

### 5.1. Inhibitors of c-KIT/PI3K/Akt/mTOR

Given that ovarian tumors express high levels of stem cell factor (KIT ligand) [35], inhibition of the c-KIT/PI3K/Akt/mTOR pathway may reduce IDO expression, and may also limit signaling through CREB, STAT3 and HIF-1α (Figure 1), all of which potentially contribute to immune suppression and disease progression. Imatinib mesylate (Gleevec) binds BCR-ABL and c-KIT, and is an effective treatment for BCR-ABL^+^ chronic myeloid leukemia. More recent studies have shown that the therapeutic effect of imatinib could also be attributed to immune response, overcoming tumor-associated T cell tolerance and enhancing vaccine efficacy [66]. Imatinib also decreased Treg frequencies and enhanced anti-tumor immune responses to DC vaccination against imatinib-resistant BCR-ABL-negative lymphoma [67], and was subsequently shown to activate CD8+ T cells and induce Treg apoptosis in a gastrointestinal tumor model through c-KIT inhibition and diminished IDO expression [31]. Stem cell factor is anti-apoptotic and increases cisplatin resistance, whereas imatinib induces apoptosis [68]. Although imatinib has shown limited clinical benefit as a single agent in ovarian cancer [69,70], it is well tolerated, and its ability to inhibit c-KIT and block IDO expression [31] suggests imatinib has potential to alleviate suppression as an adjuvant to tumor vaccination.

Sunitinib is an inhibitor of VEGFR, PDGFR, c-KIT and Flt-3, and is FDA-approved for metastatic renal cell cancer. Sunitinib is currently being tested in over 300 clinical trials for cancer treatment [71], including ovarian cancer [72,73]. Numerous studies have shown that sunitinib can reduce myeloid suppressor cell accumulation, decrease PD-L1 expression and decrease Treg frequencies in animal models [74,75] and in renal cell carcinoma patients [76,77]. This activity may at least in part be mediated through c-KIT and/or STAT3 signaling [74,75].

Downstream of c-KIT, MK-2206 and ipatasertib/GDC-0068 are potent and highly selective pan-AKT inhibitors, and both are currently in multiple phase 2 trials (clinicaltrials.gov NCT01802320, NCT02162719, NCT01776008), including treatment of ovarian cancer (NCT01283035). Specific inhibition of CREB is more problematic, since most small molecule inhibitors target CREB-binding protein, which has multiple interactions and, thus, specificity for CREB is lacking. There are several clinically approved drugs that target mTOR, notably temsirolimus, which has been reported to boost the efficacy of tumor vaccines [78]. Of particular interest, the ability of imatinib to inhibit IDO expression can be reproduced by mTOR blockade with rapamycin [31], and HIF-1α expression is also regulated at least in part through mTOR [79,80].

### 5.2. Inhibition of IL-6 and STAT3 Signaling

As IL-6 is central to the autocrine signaling loop that contributes to IDO expression, inhibition of IL-6 may have therapeutic benefit in ovarian cancer. Treatment with the anti-IL-6 antibody siltuximab inhibited IL-6 signaling, STAT3 phosphorylation, tumor growth and macrophage infiltration in ovarian cancer xenografts, and siltuximab treatment in a phase I clinical trial in ovarian cancer patients correlated with reduced plasma levels of IL-6-regulated chemokines [81]. STAT3 would appear to be the most obvious choice of drug target, given that STAT3 signaling can have a profound influence on the immunosuppressive function of tumor-associated macrophages. STAT3 signaling has also been widely implicated in tumor progression and tumor cell stemness, not least through induction of HIF-1α and Notch1 (Figure 1). Although STAT3 inhibitors are widely used experimentally, clinical application has proven to be problematic. Recent clinical trials have reported lack of clinical efficacy, poor pharmacokinetic profiles and a high incidence of adverse events, including nausea, vomiting, diarrhea and fatigue [82,83]. In a creative effort to circumvent these problems, statins have been considered as surrogate inhibitors of STAT3 signaling. Statins inhibit 3-hydroxy-3-methyl-glutaryl-CoA reductase (HMGCR), which is responsible for the synthesis of cholesterol, but they can also inhibit JAK/STAT signaling and STAT3 phosphorylation [84,85]. Simvastatin is currently being tested in combination with topotecan and cyclophosphamide for treatment of pediatric solid tumors (Clinicaltrials.gov NCT02390843), but there are no current clinical trials for ovarian cancer. In a retrospective analysis of a cohort of 442 ovarian cancer patients, there were no significant differences in progression-free survival or disease-free survival between those patients that did or did not use statins, but a secondary analysis revealed a decreased risk of disease recurrence and disease-specific death among patients with non-serous papillary types of ovarian cancer [86]. However, this study was retrospective, the number of patients with hyperlipidemia who used statins was small, and non-serous-papillary cases constitute a minor subset of the overall patient population, so the rationale for statin therapy in ovarian cancer is not strong. On the other hand, the potential for inhibition of STAT3 activation and immune modulation suggests possible adjuvant use of statins with tumor vaccines.

### 5.3. Inhibition of Wnt/β-Catenin and p38 Signaling

The wnt/β-catenin pathway is widely known to contribute to tumor progression, and may also contribute to regulation of immune suppression by macrophages/DC in the ovarian tumor microenvironment, particularly with respect to IDO expression (see Section 3.3). This pathway may thus be an attractive therapeutic target for adjuvant treatments in conjunction with tumor vaccination. LGK974 (also known as WNT974) is a novel inhibitor of Porcupine, which is an *O*-acyltransferase responsible for palmitoylation of wnt ligands, which is required for wnt secretion [87]. LGK974 is currently the subject of a phase I clinical trial for treatment of malignancies thought to be sensitive to wnt inhibition (NCT01351103). A second small molecule drug, PRI-724, blocks wnt signaling through antagonism of β-catenin/TCF-mediated transcription, resulting in down-regulation of β-catenin/TCF-responsive genes [88], potentially including IDO in tumor-associated macrophages/DC. PRI-724 is currently being tested in multiple clinical trials, including a phase I study for advanced solid tumors (NCT01302405) and a phase I/II study for treatment of myeloid malignancies (NCT01606579).

Inhibition of p38 MAPK may also be an attractive drug target, given that p38 is known to interact with wnt/β-catenin signaling, and p38 inhibition strongly ablates IDO function in human DC [58]. There are numerous clinical trials currently in progress to test the safety and therapeutic potential of p38 inhibitors in various disease settings, including a phase I/II trial of LY2228820 [89] for treatment of ovarian cancer (NCT01663857).

### 5.4. Direct Inhibition of IDO Function

This review presents many possible pathways by which IDO expression and function may be regulated in myeloid cells, including ovarian tumor-associated macrophages. Elucidation of which pathways are operative may afford opportunities for therapeutic intervention to block IDO expression. Drugs that block c-KIT, wnt, STAT3 or p38 MAPK signaling may inhibit IDO expression and may consequentially inhibit CD14+ cell-mediated immune Treg recruitment, shifting the balance in favor of Th17 responses. Given the existence of potential redundancies in pathways that induce IDO expression, a more direct approach to IDO inhibition may be advisable. Competitive inhibition of IDO with 1-methyl-d-tryptophan (1-MT) has been widely used in preclinical studies, and has been shown to suppress progression of IDO-expressing ovarian cancer cells in a syngeneic mouse model [90]. 1-MT (also known as NLG8189 or Indoximod, NewLink Genetics, Ames, IA, USA) has completed Phase I clinical testing [91] and is currently in Phase II clinical trials. A novel IDO inhibitor (NLG919 NewLink Genetics) has been described [92], and has entered clinical trials in 2013 (NCT 02048709). A third small molecule inhibitor of IDO (INCB024360, Incyte Corp.,Wilmington, DE, USA) has shown *in vivo* activity and limitation of tumor growth in animal models [93,94], and is being tested as monotherapy for ovarian cancer in a phase II clinical (NCT01685255), further underlining the translational value of IDO antagonists.

## 6. Conclusions

In contrast with the multiple mechanisms of immune suppression that prevail in the ovarian tumor microenvironment, Th17 infiltration has been associated with markedly prolonged overall survival in ovarian cancer patients [95]. This key observation has led to the formulation of a Th17-inducing DC vaccine [58] that is currently being tested in a phase I clinical trial (NCT02111941). While it makes sense to develop a tumor vaccine that boosts an immune correlate known to be associated with improved outcomes in ovarian cancer, local immune suppression will continue to present a formidable barrier to achieving clinical efficacy [35]. This review provides a roadmap of signaling circuitry centered on tumor-associated macrophages, which most likely form the fulcrum of immune regulation in ovarian cancer. The purpose is to identify checkpoints that are amenable to drug intervention at the levels of intracellular and intercellular signaling, a strategy that has clear parallels with the development of clinically successful checkpoint inhibitors that target CTLA-4 and PD-1.

Regulation of IDO expression in macrophages/DC, coupled with AhR signaling in both macrophages/DC and tumor-infiltrating T cells, is well recognized for its impact on immune regulation in cancer. Less acknowledged is the influence of wnt/β-catenin signaling, which may regulate IDO expression, and is also implicated in tumor progression. Hypoxia may also have a significant influence on macrophage/DC function, in addition to promoting cancer cell stemness and tumor survival through HIF-1α/Notch-1 signaling. All of these pathways represent attractive targets for therapeutic intervention, not least for the potential impact on immune suppression and the efficacy of tumor vaccines, but also because there may be direct benefits in limiting tumor growth.

Some pathways can be targeted by clinically approved drugs, e.g., imatinib and sunitinib inhibition of c-KIT signaling (and possibly STAT3, in the case of sunitinib), or by agents that are currently being tested in clinical trials for other indications, for example the anti-IL-6 antibody siltuximab, the AKT inhibitors MK-2206 and ipatasertib, and the wnt/β-catenin inhibitors LGK974 and PRI-724. Chief within this latter group are the second generation IDO inhibitors, which may provide a direct means of blocking a pivotal function for immune regulation in the tumor microenvironment. The search for effective inhibitors of STAT3, which is arguably at the nexus of multiple signaling pathways governing immune suppression, is altogether more problematic, and clinical trials of a leading STAT3 inhibitor (OBP-31,121) have shown a lack of efficacy accompanied by unacceptable toxicity and a high degree of pharmacokinetic variability [82,83]. Identification of AhR blockers with clinical applicability also represents a formidable challenge, given the plethora of endogenous and xenobiotic agents that are recognized by AhR, often with unpredictable agonist or antagonist actions that appear to depend on context as much as the identity of the agent. Notwithstanding this challenge, several high affinity AhR antagonists that may hold promise for future clinical trials have been identified, although none have reached clinical testing to date [96].

What are the best options for therapeutic intervention? Given the complexity and probable multiple redundancies built into signaling pathways that drive a tumor-associated macrophage M2 phenotype, it is unlikely that signal agent blockade of any particular function will be sufficient to alleviate immune suppression in the tumor microenvironment, although several options may hold potential, notably inhibition of STAT3 and wnt/β-catenin signaling. Based on current knowledge and clinical practicability, direct inhibition of IDO as an adjuvant for ovarian cancer tumor vaccination holds the greatest appeal.
